# Fragmented QRS and subclinical left ventricular dysfunction in individuals with preserved ejection fraction: A speckle‐tracking echocardiographic study

**DOI:** 10.1002/joa3.12284

**Published:** 2019-12-03

**Authors:** Mohammad Reza Dehghani, Alireza Rostamzadeh, Ali Abbasnezhad, Akram Shariati, Saeid Nejatisafa, Yousef Rezaei

**Affiliations:** ^1^ Department of Cardiology Urmia University of Medical Sciences Urmia Iran; ^2^ Heart Valve Disease Research Center Rajaie Cardiovascular Medical and Research Center Iran University of Medical Sciences Tehran Iran

**Keywords:** fragmented QRS, global longitudinal strain, normal population, speckle‐tracking echocardiography

## Abstract

**Introduction:**

Fragmented QRS (fQRS) complex on routine 12‐lead electrocardiogram (ECG) predicts adverse outcomes in patients with cardiovascular diseases. In addition, it has been found to be associated with subclinical myocardial dysfunction in chronic diseases. We sought to investigate the relationship between the presence of fQRS with the myocardial functions in individuals free from known systemic cardiovascular diseases.

**Methods:**

In a case‐control study, we evaluated normal individuals from March 2017 to February 2018. All participants underwent a 2‐dimensional transthoracic echocardiographic examination using tissue Doppler imaging (TDI) and speckle‐tracking echocardiography. In addition, all participants were examined using a 12‐lead surface ECG, and patients with fQRS and a group of age‐ and sex‐matched controls without fQRS were enrolled in our study.

**Results:**

The patients' mean age was 40.3 ± 10.7 and 35.4 ± 11.2 years in fQRS‐positive and fQRS‐negative groups, respectively (*P* = .110). Patients with fQRS had significantly lower values of apical left ventricular global longitudinal strain (LV GLS) in 2‐chamber (16.9 ± 2.5 vs. 20.5 ± 3.3, *P* < .001), 4‐chamber (16.9 ± 3.4 vs. 20.1 ± 3, *P* = .001), LAX views (17.7 ± 2.8 vs. 20.8 ± 3.5, *P* = .001), and averaged LV GLS (17 ± 2.6 vs. 20.4 ± 2.7, *P* < .001) values compared to patients without fQRS. In a multivariate analysis, averaged LV GLS and smoking history were independent predictors for positive fQRS.

**Conclusion:**

The presence of fQRS on 12‐lead ECG in healthy population was associated with lower values of LV GLS compared to normal individuals without fQRS.

## Graphical abstract

The presence of fragmented QRS complex on standard 12‐lead electrocardiogram in healthy individuals is associated with lower values of left ventricular global longitudinal strains compared to individuals without fragmented QRS complex, indicating a subclinical myocardial dysfunction.

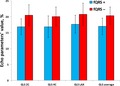

## 1 INTRODUCTION

Fragmented QRS (fQRS) complex is detected in a 12‐lead electrocardiogram (ECG), which has enormously been used for the prediction of cardiovascular outcomes.^1^ The fQRS indicates altered ventricular depolarization caused by myocardial fibrosis and scar in patients with ischemic heart diseases.^2^, ^3^, ^4^, ^5^ It is not only observed in patients with coronary artery diseases, but is also found in cardiomyopathies, structural heart diseases, heart rhythm disturbances, cardiac sarcoidosis, and even healthy population.^1^, ^6^, ^7^, ^8^ The fQRS has been found to be a marker of clinical outcomes in different cardiovascular diseases. Several studies have shown that the fQRS is a predictor of sudden cardiac death in patients with cardiomyopathy and heart failure,^6^, ^9^ a predictor of cardiac events and mortality in patients with coronary artery disease,^10^, ^11^, ^12^ a marker for nonresponsiveness to cardiac resynchronization therapy in heart failure patients with ventricular dyssynchrony,^13^ a prognostic marker for dysrhythmia in patients with Brugada syndrome,^14^ and short‐term prognosis of patients undergoing transcatheter aortic valve implantation.^15^

The standard 12‐lead ECG can be suggestive of abnormal findings during echocardiographic findings and patients should be regularly followed up.^16^ In addition to the prognostic value of fQRS in patients with cardiovascular diseases, it has also been found to be associated with subclinical myocardial dysfunction in patients with coronary artery disease,^17^ diabetes mellitus,^18^ and chronic kidney disease.^19^ The assessment of myocardial tissue by conventional echocardiography is load‐ and angle‐dependent, but 2‐dimensional speckle‐tracking echocardiography (2D‐STE) lacks such a limitation for the evaluation of myocardial mechanics.^20^, ^21^, ^22^, ^23^ Although left ventricular ejection fraction (LVEF) is commonly used for the evaluation of LV function, 2D‐STE parameters, mainly global longitudinal strain (GLS), have been found to be a feasible modality for examination of left ventricle (LV) function.^24^

In this case‐control study, we sought to determine the relationship between the presence of fQRS in apparently healthy individuals without cardiovascular diseases and preserved LVEF and subclinical LV dysfunction using 2‐dimensional transthoracic echocardiography (2D‐TTE) with the implementation of tissue Doppler imaging (TDI) and speckle‐tracking echocardiography.

## 2 METHODS AND MATERIALS

### 2.1 Study protocol and population

In a case‐control study, we prospectively enrolled normal individuals with or without fQRS on their 12‐lead surface ECG at rest, from March 2017 to February 2018 in Urmia city, Iran. All participants underwent a comprehensive 2D‐TTE examination using TDI and STE modalities to evaluate the structural and functional features of their heart. The study was approved by the institutional review board in Seyyed‐al‐Shohada Heart Center and the local ethics committee of Urmia University of Medical Sciences, Urmia, West Azerbaijan province, Iran. In addition, informed consent was obtained from all participants at baseline.

Twenty‐six consecutive patients who visited our outpatient clinic with positive fQRS were recruited into this study. In addition, 28 age‐ and sex‐matched individuals who had negative fQRS in baseline ECG were also enrolled. Inclusion criteria included healthy individuals who were referred to be evaluated for probable cardiac diseases before military service entrance, employment in governmental organizations, a requirement for insurance validity, and participation in sport events. In addition, some people who visited our outpatient clinic for palpitation and atypical chest pain with a low probability of coronary artery diseases whose stress tests and/or cardiac scans had been negative for any ischemic heart diseases. Exclusion criteria included patients with known history of coronary artery diseases, any signs of ischemic heart diseases in stress tests and cardiac scan, heart failure, diabetes mellitus, hypertension, hypercholesterolemia, LVEF lower than 55%, any significant valvular diseases (ie, greater than mild involvement), any congenital heart diseases, atrioventricular conduction abnormalities, QRS duration longer than 120 milliseconds, pulmonary arterial hypertension, chronic obstructive pulmonary diseases, consumption of any medications for chronic diseases, and bundle branch block in ECG trace.

### 2.2 Electrocardiographic examination

All participants were examined by a 12‐lead surface ECG at rest (0.5 Hz to 150 Hz, 25 mm/s, 10 mm/mV). ECGs were analyzed by two clinicians who were blinded to the impression of cases. In case of disagreement, the mutual agreement was used to reach a final diagnosis. The fQRS was defined as the detection of RSRʹ pattern, which can be observed as follows: (i) an additional R wave; (ii) a notching in nadir of the S wave; (iii) a notching of the R wave; (iv) Rʹ wave >1 millimeter; and (v) the presence of fragmentation, more than one Rʹ wave, in two contiguous ECG leads.^1^, ^25^

### 2.3 Echocardiographic examination

All echocardiographic measurements were performed based on the recommendations of the American Society of Echocardiography and the European Association of Cardiovascular Imaging,^26^ using an ultrasound scanner (Vivid S6). A 2D‐TTE was used to evaluate the presence of any structural heart diseases, valvular and congenital heart diseases. The LVEF was also measured using the biplane Simpson's method. All echocardiographic examinations were carried out by a single echocardiographer in our echocardiography laboratory.

A standard M‐mode, 2D, and color‐coded TDI images were obtained during breath hold, as an average of three consecutive beats. The TDI was provided from the annular and septal mitral valve area. The harmonic image recordings of apical and short‐axis views at mitral valve and papillary muscle levels (30‐90 frames/second) were provided and stored to be analyzed offline using AFI (automated functional imaging) software. The left ventricular GLS (LV GLS) was calculated by averaging apical 4‐chamber, 2‐chamber, and LAX views. Based on a meta‐analysis,^27^ the ranges of GLS in normal population is 15.9% to 22.1%; therefore, the GLS was considered abnormal when it was smaller than 16 in our cohort. The evaluation of LV diastolic dysfunction was also performed using the latest American Society of Echocardiography guideline for LV diastolic dysfunction as well.^28^

### 2.4 Statistical analysis

All variables are presented as mean ± standard deviation, and *t* test was used to compare continuous variables between groups. Chi‐squared tests or Fisher's exact test was implemented for comparing categorical variables as appropriate. A multivariate regression analysis was conducted to evaluate the relationship between fQRS and LV GLS values. The model was also adjusted for age, gender, smoking history, QRS duration, QT interval corrected for heart rate, body mass index, and total cholesterol level. Adjusted odds ratios (ORs) and corresponding confidence intervals (CIs) were reported. A two‐tailed *P* < .05 was considered statistically significant. All statistical analyses were performed using the IBM SPSS software version 22.0 (IBM, Armonk).

## 3 RESULTS

The patients' mean age was 40.3 ± 10.7 and 35.4 ± 11.2 years in fQRS‐positive and fQRS‐negative groups, respectively (*P* = .110). The number of smokers was significantly higher in positive fQRS compared to negative fQRS group (30.8% vs 7.1%; *P* = .026). The half of patients with fQRS (13%, 50%) had fQRS in less than three leads and others had fQRS in three or more than three leads. Fifteen patients (57.7% of fQRS patients) had fQRS in inferior leads, five patients (19.3% of fQRS patients) in precordial leads, and others (23% of fQRS patients) in a combination of precordial and limb leads. The QRS duration was comparable between individuals with or without fQRS (93.5 ± 6.3 and 93.6 ± 6.2 msec, respectively; *P* = .949). All laboratory data and other parameters of ECG were comparable between groups (Table 1).

**Table 1:** Baseline characteristics in patients with or without fQRS

	Positive fQRS (n = 26)	Negative fQRS (n = 28)	*P* value
Age, year	40.3 ± 10.7	35.4 ± 11.2	.110
Male	21 (80.8%)	17 (60.7%)	.107
BMI, kg/m2	26.3 ± 4.7	26.3 ± 3.3	.989
Systolic BP, mm Hg	118.5 ± 10.5	119 ± 9.8	.836
Diastolic BP, mm Hg	70.4 ± 7.3	71 ± 8.6	.793
Smoking	8 (30.8%)	2 (7.1%)	.026
Familial history of CVD	4 (15.4%)	1 (3.6%)	.135
ECG parameters			
Heart rate, bpm	76.5 ± 9.4	78.2 ± 8.8	.493
P duration, ms	85.26 ± 5.8	84.8 ± 6.7	.793
P amplitude, ms	1.9 ± 0.3	2 ± 0.2	.302
PR interval, ms	150.4 ± 23.2	154.6 ± 24.1	.512
QRS duration, ms	93.5 ± 6.3	92.5 ± 7	.599
QTc, ms	393.7 ± 17.9	391.5 ± 21.5	.682
Laboratory parameters			
Potassium, mg/dl	4.1 ± 0.4	4.2 ± 0.4	.576
Creatinine, mg/dl	1 ± 0.15	1 ± 0.2	.304
Fasting blood glucose, mg/dl	90.3 ± 9.9	90.5 ± 8.4	.951
Cholesterol, mg/dl	178.5 ± 42.1	194.6 ± 27	.105
Triglyceride, mg/dl	219.5 ± 42.3	208.3 ± 28.6	.259

Note

Data are presented as mean ± SD or number (%).

All comparisons are calculated using Chi‐squared test or *t* test.

Abbreviations: BMI, body mass index; BP, blood pressure; bpm, beat per minute; CVD, cardiovascular diseases; ECG, electrocardiogram; fQRS, fragmented QRS; ms, millisecond; QTc, QT interval corrected for heart rate.

**Figure 1:**
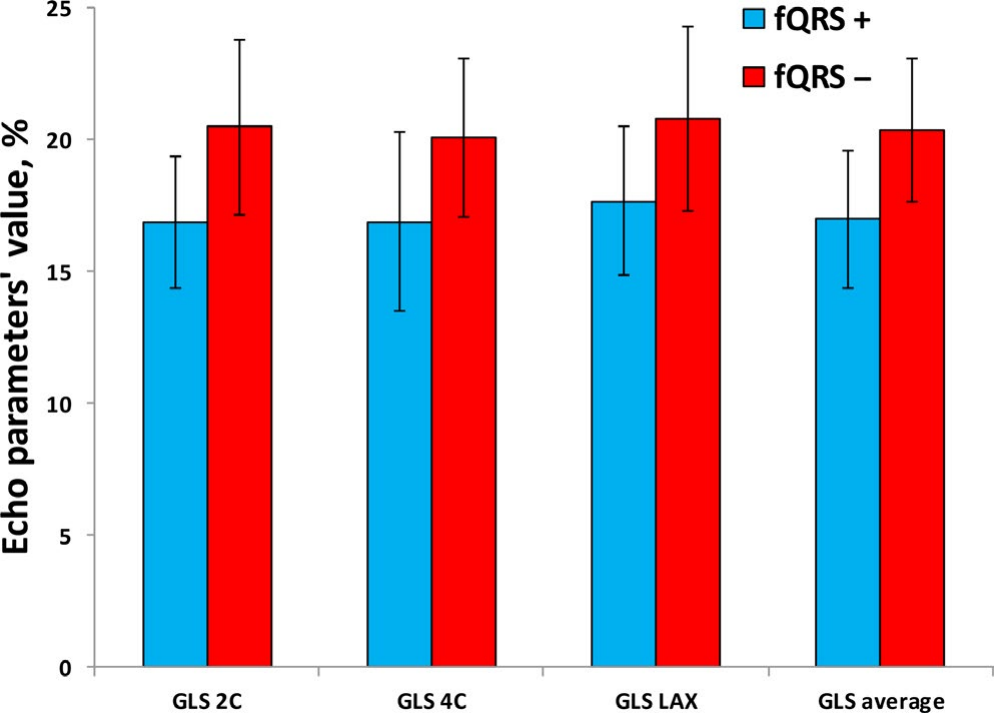
Speckle‐tracking echocardiographic parameters in patients with or without fragmented QRS

When comparing echocardiographic parameters, the LVEF was comparable between groups (59.6 ± 2.4% vs 58.9 ± 2.5%, *P* = .310). However, based on 2D‐STE, patients with fQRS had significantly lower values of apical LV GLS in 2‐chamber (16.9 ± 2.5 vs 20.5 ± 3.3, *P* < .001), 4‐chamber (16.9 ± 3.4 vs 20.1 ± 3, *P* = .001), LAX views (17.7 ± 2.8 vs 20.8 ± 3.5, *P* = .001), and averaged LV GLS (17 ± 2.6 vs 20.4 ± 2.7, *P* < .001) values compared to individuals without fQRS (Figure 1). The number of individuals with abnormal LV GLS (defined as <16) was significantly higher in individuals with fQRS compared to those without fQRS (23.1% vs 0%, *P* = .009). In addition, there was no significant difference between groups regarding the number of individuals with mild LV diastolic dysfunction (7.7% vs 10.7%, *P* = .626). Other echocardiographic values are summarized in Table 2.

**Table 2:** Echocardiographic measurements in patients with or without fQRS

	Positive fQRS (n = 26)	Negative fQRS (n = 28)	*P* value
LVEF, %	59.6 ± 2.4	58.9 ± 2.5	.310
LVEDV, mL	95.6 ± 17.6	87.6 ± 18.2	.109
LVESV, mL	41.2 ± 6.5	38.5 ± 8.2	.192
LVEDD, mm	4.8 ± 0.3	4.7 ± 0.4	.106
IVSD, mm	1 ± 0.1	0.9 ± 0.08	.008
PWD, mm	0.9 ± 0.08	0.85 ± 0.09	.009
LA volume, mL	37.4 ± 8	36.7 ± 8.2	.775
LVH	4 (14.3%)	5 (19.2%)	.626
E, cm/s	0.7 ± 0.06	0.7 ± 0.08	.573
A, cm/s	0.5 ± 0.07	0.5 ± 0.09	1
E/A	1.3 ± 0.2	1.3 ± 0.3	.957
eʹ, cm/s	1.1 ± 0.08	1 ± 0.09	.114
E/eʹ	1.2 ± 0.1	1.2 ± 0.1	.393
DT, ms	228.8 ± 21.1	229.6 ± 21.6	.892
RVOT VTI, cm	18.5 ± 1.3	18.5 ± 1.7	.933
LVOT VTI, cm	21.8 ± 1.9	21.9 ± 2.5	.895
Aortic root size, cm	3.2 ± 0.2	3.1 ± 0.3	.266
TR velocity, m/s	2.1 ± 0.2	2.1 ± 0.1	.649
PAP, mm Hg	21 ± 3.1	20.6 ± 2.2	.596
LVDD*	2 (7.7%)	3 (10.7%)	.626
GLS 2‐chamber, %	16.9 ± 2.5	20.5 ± 3.3	<.001
GLS 4‐chamber, %	16.9 ± 3.4	20.1 ± 3	.001
GLS LAX, %	17.7 ± 2.8	20.8 ± 3.5	.001
Averaged GLS, %	17 ± 2.6	20.4 ± 2.7	<.001
Abnormal GLS, values < 16	6 (23.1%)	0 (0)	.009

Note

Data are presented as mean ± SD or number (%).

All comparisons are calculated using Chi‐squared test, *t* test, or Fisher's exact test.

Abbreviations: A, peak late diastolic mitral velocity; DT, deceleration time; E, peak early diastolic mitral velocity; eʹ, peak early velocity; GLS, global longitudinal strain; IVSD, interventricular septum diameter; LA, left atrium; LVDD, left ventricular diastolic dysfunction; LVEDD, left ventricular end‐diastolic diameter; LVEDV, left ventricular end‐diastolic volume; LVEF, left ventricular ejection fraction; LVESV, left ventricular end‐systolic volume; LVOT, left ventricular outflow tract; PAP, pulmonary arterial pressure; PWD, posterior wall diameter; RVOT, right ventricular outflow tract; VTI, velocity time integral.

* All patients had grade I diastolic dysfunction.

Due to a high number of smokers among individuals with fQRS, we compared echocardiographic values among individuals with or without fQRS in subgroups by smoking history. All echocardiographic parameters were comparable between smoker and nonsmokers too (data are not reported). When compared individuals with fQRS in less than three leads and those with fQRS in more than three leads, the LV GLS value was comparable between both groups (data not reported).

In multivariate analysis, averaged LV GLS (OR 0.426, 95% CI 0.241 ‐ 0.751, *P* = .003) and smoking history (OR 18.033, 95% CI 1.086 ‐ 299.404, *P* = .044) were independent predictors for positive fQRS. Other data are summarized in Table 3.

**Table 3:** Univariate and multivariate analyses for association between GLS and fQRS

	Odds ratio	95% confidence interval	*P* value
Univariate analysis			
Averaged GLS	0.525	0.352 ‐ 0.783	.002
Multivariate analysis			
Averaged GLS	0.426	0.241 ‐ 0.751	.003
Age	1.055	0.967 ‐ 1.151	.225
Male	0.745	0.104 ‐ 5.346	.769
BMI	1.081	0.886 ‐ 1.319	.445
QRS duration	1.154	0.990 ‐ 1.344	.066
QTc	1.019	0.974 ‐ 1.067	.419
Smoking	18.033	1.086 ‐ 299.404	.044
Total cholesterol	0.989	0.967 ‐ 1.012	.348

Abbreviation: GLS, global longitudinal strain.

## 4 DISCUSSION

In this case‐control study, we showed that among apparently healthy individuals with normal findings in routine cardiovascular screening using a 12‐lead standard ECG and a 2D‐STE, the LV GLS values were significantly lower in individuals with fQRS than those without fQRS. Although those with fQRS smoked more than non‐fQRS participants, there was no significant difference with regard to the echocardiographic parameters between individuals with or without smoking history. In addition, on multivariate analysis, averaged LV GLS, inversely, and smoking, directly, were associated with the presence of fQRS in our cohort.

Cardiac magnetic resonance imaging and histopathologic evaluations of the myocardium can be used for detecting myocardial fibrosis and/or scar;^29^, ^30^ however, these modalities are invasive or expensive for the assessment of cardiac tissue. In contrast, the fQRS as a readily available, noninvasive, and low‐cost tool may provide some clues for the evaluation of myocardial scar. Uslu et al^12^ retrospectively evaluated individuals with myocardial infarction and found that LV wall motion, measured by echocardiography, was significantly associated with the presence of fQRS. Das et al^4^ also showed that the fQRS, in the presence of wide QRS complex, was a relatively sensitive and highly specific marker for identifying myocardial scar in patients with known or suspected coronary artery disease. In addition, in a meta‐analysis of 2026 patients with myocardial infarction,^31^ the fQRS at baseline predicted the development of LV dysfunction, major cardiovascular events, and mortality.

The fQRS has also been found to be associated with subtle myocardial dysfunction in several reports. Oner et al^32^ evaluated patients with metabolic syndrome and compared them with matched control individuals without metabolic syndrome. They observed that fQRS was more common among patients with metabolic syndrome compared to those without it (26.1% vs 14.6%, *P* = .041). In addition, when compared to healthy controls, metabolic syndrome patients with fQRS had a higher E/e' and lower e' velocity, indicators of diastolic dysfunction, as well as lower isovolumic acceleration, indicating subclinical LV systolic dysfunction. In addition, E/e' and isovolumic acceleration were independent predictors of fQRS in patients with metabolic syndrome. Yan et al^17^ evaluated 176 patients with coronary artery disease with preserved LVEF (>45%). After examination of all patients using 2D‐STE, they found that global, middle, and apical LV longitudinal, radial, and circumferential strains and strain rates were significantly lower in the fQRS group compared to the non‐fQRS. Adar et al^19^ evaluated the ventricular functions in patients with chronic kidney disease who had a normal LVEF (≥50%). The prevalence of fQRS was significantly higher in patients with an abnormal Tei index, indicating LV dysfunction, compared to patients with a normal Tei index (71% vs 40%). Moreover, those with an abnormal Tei index had a lower E/A compared to those with a normal Tei index. These findings are in lines with our findings showing the lower levels of LV GLS in patients with fQRS, but in a healthy population.

Based on a population‐based study in Finland,^8^ the rate of healthy individuals with fQRS was 18.3% (1518 of 8277 individuals without known cardiac diseases), and it has been found to be 14.6% among a healthy population in a case‐control study.^32^ In contrast, the prevalence of fQRS in other population is significantly greater than healthy population. The prevalence of fQRS in patients with chronic kidney disease with preserved LVEF,^19^ metabolic syndrome,^32^ heart failure patients requiring resynchronization therapy,^13^ and diabetes mellitus^18^ was 60%, 26%, 41.5%, and 28%, respectively. In a former population‐based study, the fQRS in healthy middle‐aged individuals was frequent, but it did not affect the occurrence of cardiac death, arrhythmic death, and death from any causes during a mean of 30 years follow‐up.^8^ However, among individuals with evidences of cardiac diseases, the fQRS in lateral leads was associated with adverse events. In addition, in a recently published case‐control study, Yaman et al^33^ found that LV GLS, reflecting systolic function, and E/A, reflecting diastolic function, were lower in individuals with fQRS compared to those without fQRS. Epicardial adipose tissue thickness was also significantly increased in fQRS‐positive participants.

In our study, the majority of participants were young and all individuals were healthy without cardiovascular diseases on routine screening; however, there was significant subclinical myocardial dysfunction in the echocardiographic examination. Similar to Yaman et al 33 study, we found that healthy individuals with fQRS had LV systolic dysfunction measured by LV GLS, while there was no difference in LV diastolic dysfunction. We did not follow‐up participants, and so we could not conclude regarding the prognostic value of fQRS in the healthy population with or without abnormal LV GLS values. On the other hand, in a recently published results of the Copenhagen city heart study,^34^ low‐risk general population with a decreased GLS at baseline experienced worse outcomes compared to those with higher values of LV GLS during a median of 11 years follow‐up (hazard ratio 1.12; 95% confidence interval 1.08 to 1.17; *P* < .001 per 1% decrease in GLS levels). Moreover, on multivariate analysis, this association remained significant even after adjustment for other factors; however, gender modified this association, so that GLS did not predict outcomes in women rather than men. The present study was a case‐control study and participants were matched by sex and age, so we could not find any effect of sex on our findings. Given previous studies and our study, we believe that it can be postulated that among apparently healthy population, the presence of fQRS might be considered as a surrogate of subclinical myocardial dysfunction, which can be evaluated by 2D‐STE. The impact of this association on the outcomes of apparently healthy population warrants further large‐scale studies to assess whether concomitant fQRS and decreased LV GLS in a healthy population are associated with worse outcomes at long‐term follow‐up or not.

### 4.1 Study limitations

This study suffers from some shortcomings needed to be addressed in future studies. Firstly, it was a case‐control study with small sample size. Secondly, all participants with atypical chest pain were categorized as low‐risk group and they did not undergo coronary angiography, so we could not definitely say that all patients were free of coronary artery disease. Thirdly, we performed neither noninvasive imaging for the evaluation of myocardial scar/fibrosis nor a pathologic evaluation of myocardial tissue to show that which pathogenesis might contribute to such a phenomenon in our population, fQRS and lower LV GLS. Finally, we did not follow‐up individuals to pursue the prognostic value of concomitant fQRS and decreased LV GLS in the healthy population. Given these limitations in this study, it seems that future studies should focus on these issues.

## 5 CONCLUSION

The presence of fQRS on standard 12‐lead ECG in the apparently healthy population is significantly associated with lower values of LV GLS compared to individuals without fQRS, indicating a subclinical myocardial dysfunction.

## CONFLICT OF INTEREST

The authors declare no conflict of interests for this article.
